# Intranasal vaccination with M2e5x virus-like particles induces humoral and cellular immune responses conferring cross-protection against heterosubtypic influenza viruses

**DOI:** 10.1371/journal.pone.0190868

**Published:** 2018-01-11

**Authors:** Young-Tae Lee, Eun-Ju Ko, Youri Lee, Ki-Hye Kim, Min-Chul Kim, Yu-Na Lee, Sang-Moo Kang

**Affiliations:** 1 Center for Inflammation, Immunity & Infection, Institute for Biomedical Sciences, Georgia State University, Atlanta, Georgia, United States of America; 2 Animal and Plant Quarantine Agency, Gimcheon, Gyeongsangbukdo, Republic of Korea; University of Georgia, UNITED STATES

## Abstract

Current influenza vaccines do not provide broad cross-protection. Here, we report that intranasal vaccination with virus-like particles containing the highly conserved multiple ectodomains of matrix protein 2 (M2e5x VLP) of influenza virus induces broad cross-protection by M2-specific humoral and cellular immune responses. M2e5x VLP intranasal vaccination prevented severe weight loss, attenuated inflammatory cytokines and cellular infiltrates, and lowered viral loads, and induced germinal center phenotypic B and plasma cells. In addition, depletion studies demonstrate the protective roles of CD4 and CD8 T cells induced by M2e5x VLP intranasal vaccination. Thus, this study provides evidence that mucosal delivery of M2e5x VLP vaccine provides cross-protection by inducing humoral and cellular immune responses.

## Introduction

Influenza virus causes respiratory viral diseases in humans and animals, with significant medical and economic burdens. Approximately 250,000–500,000 deaths are estimated annually worldwide due to influenza-related disease [1, 2]. The emergence of the 2009 pandemic H1N1 virus is an example of a new strain with distinct antigenic properties by triple reassortment [3, 4]. While antibodies to hemagglutinin (HA) provide strain-specific protection, the current vaccine formulations are not effective in protection against antigenically distinct strains.

The ion-channel protein M2 has an extracellular domain of 24 amino acids (M2e) which is a conserved molecular target among human influenza A strains [5, 6]. To overcome the low immunogenicity of M2 protein, previous studies approached M2e-conjugate carrier vehicles, potent adjuvants, and multiple immunizations with high vaccine doses [7–14]. In a previous study, we generated a molecular construct containing a tandem repeat of M2 ectodomain and presented it on virus-like particles (M2e5x VLP) [15]. Intramuscular immunization with M2e5x VLP in the absence of adjuvants induced M2 specific antibodies and cross-protection [15].

Since influenza virus is a respiratory pathogen, mucosal immunization with cross protective M2e-based vaccines could be effective in conferring protection. Previous studies reported that intranasal immunization with M2e-conjugate protein vaccines with chitosan adjuvant or M2e-flagellin adjuvant chimeric on VLP resulted in survival protection despite severe weight loss [16–18]. Nasal-inactivated vaccines in clinical trials raised serum and mucosal antibodies which could be cross-protective and have the advantages of potential application to the high risk groups over live vaccines [19]. In this study, we investigated the immunogenicity and efficacy of M2e5x VLP intranasal immunization in a mouse model. Also, to better understand cross protective immune correlates after intranasal vaccination, detail systemic and local cellular immune responses including germinal center and plasma cells, and cytokine-secreting T cell responses were determined, demonstrating the effective cross protection preventing weight loss.

## Materials and methods

### Viruses and M2e5x VLP vaccines

**Fig 1:**
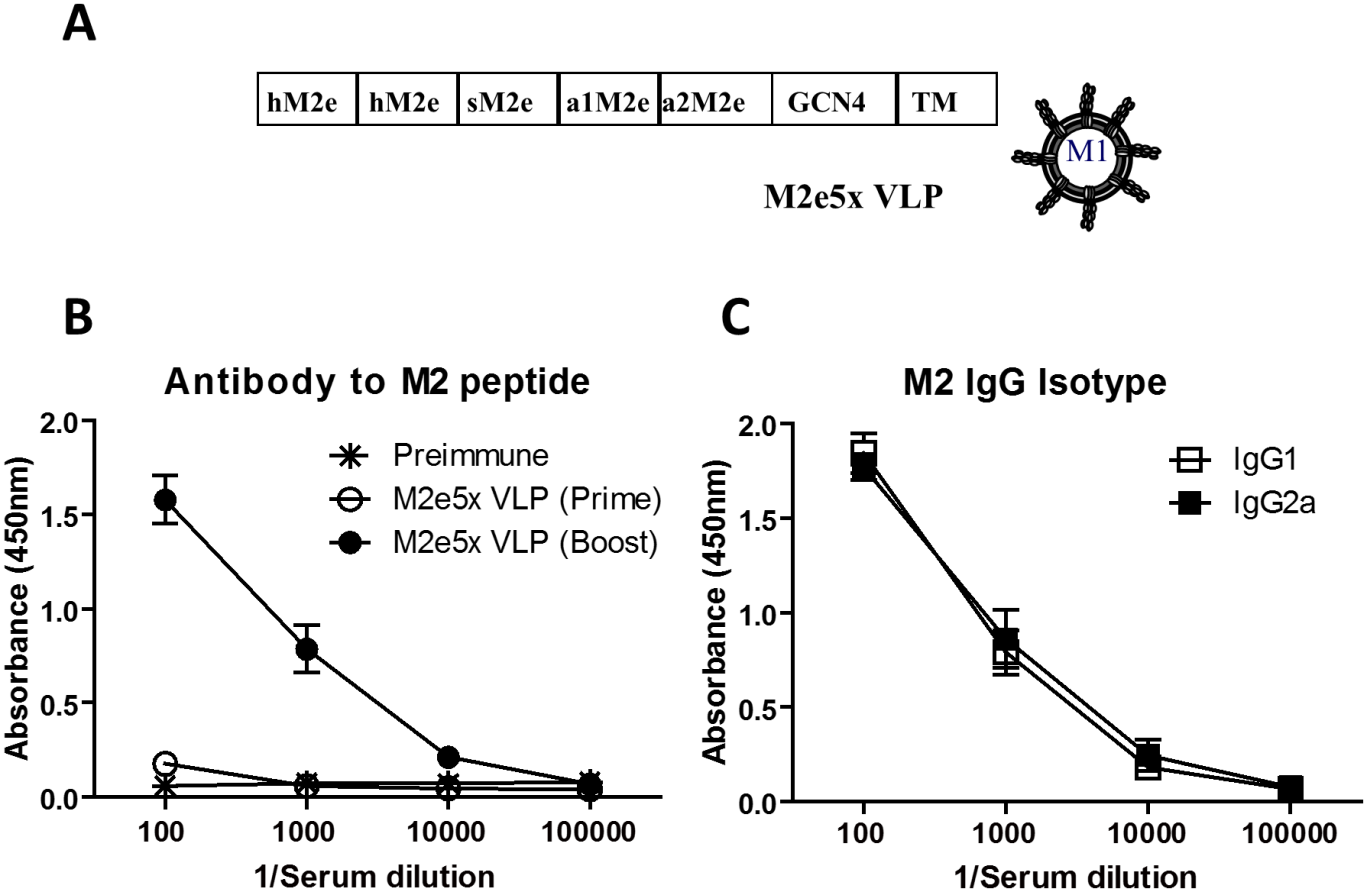
Intranasal vaccination with M2e5x VLP induces M2e specific IgG antibody responses. **(A)** Schematic diagram of M2e5x construct and M2e5x VLP. hM2e: human influenza A type M2e, sM2e: swine influenza A type M2e, a1M2e: Avian influenza A type I M2e, a2M2e: Avian influenza A type II M2e. GCN4: the oligomer-stabilizing domain of general control nondepressible 4 (GCN4) as reported [41]. TM-tail: A/PR8 virus hemagglutinin transmembrane and cytoplasmic tail domains. M1: A/PR8 virus M1 matrix protein responsible for assembling VLP structures. **(B-C)** Naïve BALB/c mice (n = 10) were intranasally immunized with M2e5x VLP (15μg/mouse) at week 0 and boosted at week 4 (Fig 2A). Antigen-specific antibody titers were measured 3 weeks after prime and boost immunization. **(B)** M2e specific IgG. Immune sera were serially diluted and IgG levels against M2e peptide were analyzed by ELISA. **(C)** Isotypes of M2 specific IgG antibodies were determined using anti-mouse IgG isotype antibodies.

Mouse adapted A/Philippines/2/1982 (A/Phil, H3N2) virus was generously provided by Dr. Huan Nguyen. Reassortant A/Viet rgH5N1 virus (rgH5N1; HA and NA were derived from A/Vietnam/1203/2004 and the backbone genes from A/PR/8/34 virus) was previously described [20]. The reassortant rgH5N1 virus has the same M2e from the M gene of A/PR8. Viruses were propagated in embryonated chicken eggs, and allantoic fluids clarified by centrifugation (3600 × g, 30 minutes [min]) and kept at -80°C. M2e5x VLP (Fig 1A) was produced using the recombinant baculovirus (rBV) expression system as previously described [15]. Briefly, to produce M2e5x VLPs, Sf9 cells were coinfected with rBVs expressing influenza M1 matrix protein and a tandem repeat of M2 ectodomains (M2e5x) derived from human (2x, SLLTEVETPIRNEWGSRSN), swine (1x, SLLTEVETPTRSEWESRSS), Avian 1 (1x, SLLTEVETPTRNEWESRSS), and Avian 2 (1x, SLLTEVETLTRNGWGCRCS) influenza viruses (Fig 1A). Culture supernatants containing M2e5x VLP were collected by centrifugation at 2,000 × g for 20 min and further spun by ultra-centrifugation at 100,000 × g for 1 hour (h). M2e5x VLP was further purified by ultracentrifugation using discontinuous sucrose gradients (20%-30%-60%). A recommended level for recombinant subunit vaccines is 20 endotoxin units (EU)/ml [21]. The endotoxin levels of prepared 5xM2e VLP vaccine were determined by Chromogenic LAL endotoxin assay kit (Cat# L00350, GenScript) and found to be less than 1.2 EU/15 μg 5xM2e VLP, which is within an acceptable level.

### Immunization and challenge

**Fig 2:**
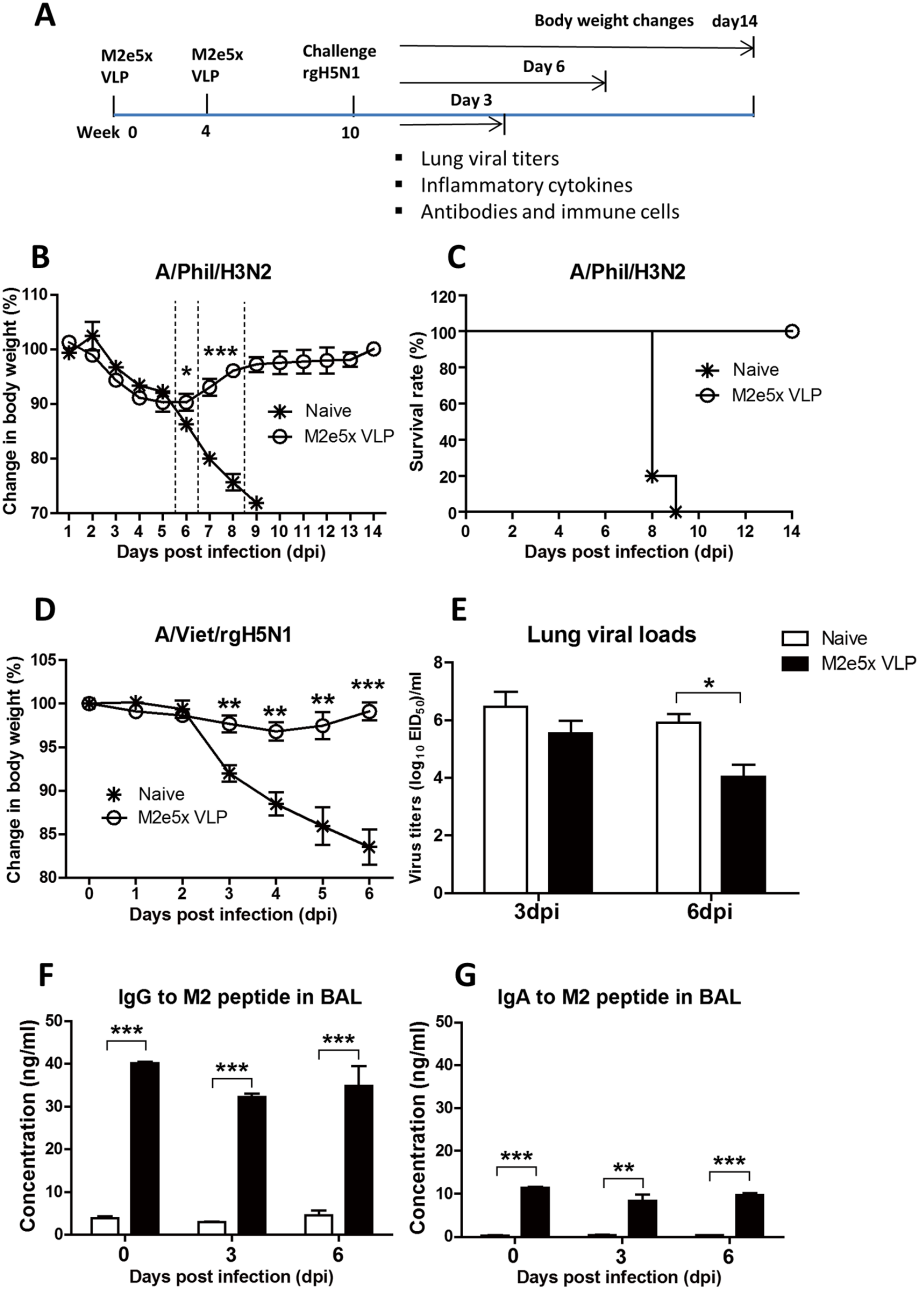
M2e5x VLP intranasal immunization induces mucosal antibodies and cross-protection preventing weight loss. **(A)** A detailed timeline for the vaccination, challenge, and sampling strategy. BALB/c mice that were intranasally immunized with M2e5x VLPs (15 μg/mouse) at weeks 0 and 4, and were challenged with different subtype influenza viruses at week 10. Day 3, 6, and 14 indicate the days when samples were analyzed or body weight changes monitored for 14 days during virus infection. **(B)** Body weight change and **(C)** survival rates after challenge with A/Phil (H3N2) (5×LD_50_). **(D)** Body weight changes after challenge with rgH5N1 (3×LD_50_) virus. **(E)** Lung viral loads at 3 and 6 dpi (days post infection) with rgH5N1 (3×LD_50_). **(F)** M2e-specific IgG antibodies in the BAL at 0, 3, and 6 dpi. **(G)** M2e-specific IgA antibodies in the BAL at 0, 3, and 6 dpi. Statistical significance was determined using an unpaired two-tailed Student’s *t* test. Error bars are means ± SEM of concentration or ratios from individual animals. *, *P*<0.05; **, *P*<0.01; ***, *P*<0.001.

Female BALB/c mice (n = 5 per group, 6- to 8-week-old) purchased from Harlan Laboratories were intranasally immunized with 15 μg of M2e5x VLP total proteins (0.9 μg M2e5x proteins) in 50 μl at weeks 0 and 4. Immune sera were collected to determine antigen-specific antibody responses 3 weeks after prime and boost immunizations. M2e5x VLP immune mice were challenged with a lethal dose (5×LD_50_) of A/Phil (H3N2) or A/Viet (rgH5N1) virus at 6 weeks after boost immunization. In separate experiments, mice were sacrificed to collect samples at day 0 (before challenge), or day 3 and 6 post infection (Fig 2A). If mice show weight loss above the endpoint (20%) compared to the control animals, then mice are euthanized immediately. The mice that show clinical signs of illness such as ruffled hair coat and/or difficult breathing are humanely euthanized to avoid the pain. The methods of euthanasia used in the study include isoflurane and carbon dioxide, and death is confirmed by cervical dislocation. Animals are anesthetized using isoflurane inhalation before the distressful procedures to relieve the pain. All animal experiments were approved and performed under the guidelines by the Georgia State University Institutional Animal Care and Use protocol (A14025).

### Humoral immune responses

The levels of M2e specific antibody titers were determined by ELISA using human type M2e peptides (SLLTEVETPIRNEWGSRSN) as a coating antigen. ELISA 96-well plates were coated with M2e peptides (4 μg/ml) overnight and then blocked with 1% bovine serum albumin (BSA) and 0.05% Tween 20 in phosphate-buffered saline (PBS) for 1.5 h at 37°C. After wash, diluted samples (sera, bronchoalveolar lavage fluid [BALF], or *in vitro* culture supernatants) were added and incubated for 1.5 h at 37°C. For determination of class-switched antibodies, the horse radish peroxidase (HRP)-conjugated goat anti-mouse IgG, IgG1, IgG2a, and IgA secondary antibodies (Southern Biotechnology) were used. The substrate of 3,3’,5,5’-tetramethylbenzidine (TMB) (Sigma Aldrich) was used to develop color and stopped with 1M H_3_PO_4_. The OD value was determined at 450nm using a BioTek ELISA plate reader. Antibody amounts were quantified using the standard curve for each IgG isotype antibody.

### T cell depletion experiments

To understand the role of CD4 T and CD8 T cells in protection, M2e5x VLP immune mice were intravenously injected with anti-CD4 (200 μg in 200 μl, Clone GK1.5, Catalog # BE003-1) and anti-CD8 (150 μg in 200 μl, Catalog # BE0061) depletion antibodies (Bio X Cell) at 2 days before infection and 1 day after infection with A/Phil (H3N2) virus. The levels of T cell depletion were confirmed by flow cytometry, resulting in depleting T cells over 99% (S1 Fig). Injection of IgG isotype control (Clone LTF-2, rat IgG2b, Cat # BE0090, BioXCell) did not affect the depletion of T cells similar to PBS. Body weight changes were monitored for 7 days. Lung viral loads were determined by measuring embryonated egg infectious unit titers at 7 days after challenge.

### Determination of antibody producing cells

To determine the levels of *in vitro* M2e specific antibody production as a measure of antibody secreting cells, the splenocytes (memory B cells) and bone marrow (plasma) cells were collected at day 6 post challenge. Million cells isolated from spleens and bone marrow were added in triplicates (10^6^ cells / 0.2 ml) to M2e peptide-coated plates and then incubated for 1 or 5 days at 37°C. The IgG antibodies secreted into supernatants after *in vitro* cultures for 1 or 5 days were determined by ELISA as described above.

### Cytokine assays

To compare inflammatory responses, proinflammatory cytokines were measured by cytokine ELISAs, performed as previously described by Lee et al. [22]. Briefly, Ready-Set-Go interferon-gamma (IFN-γ) and tumor necrosis factor alpha (TNF-α) kits (eBioscience, San Diego, CA) were used to determine the amounts of cytokines in the BALF and lung extracts following the manufacturer’s procedures.

### Cell preparation and intracellular cytokine staining analysis

Immune cells were harvested from the airways via BAL. The lung tissues were homogenized and lung extracts spun on 44/67% Percoll at 3600 × g, for 15 min. The layer containing cells between 44 and 67% Percoll gradients were collected for the analysis of lung cells. The mediastinal lymph nodes (MLNs) were grinded using the frosted glass microscope slides to make cell suspensions. For antigen-specific T cell responses, the cells from the BAL and lungs were *in vitro* stimulated with human M2 peptides (5 μg / ml) for 5 h at 37°C in the presence of Brefeldin A (BFA) (20μg/ml). After stimulation, lymphocytes were stained with CD4 (CD4-PE/Cy5, Cat# 100410, Biolegend) and CD8 (CD8α-PE, Cat# 100708, Biolegend) monoclonal antibodies. BD Cytofix/Cytoperm^™^ Plus kit was used to fix and permeabilize specific marker labelled lymphocytes and cytokine positive cells were then stained with IFN-γ cytokine antibodies (anti-mouse IFN-ɣ APC-Cy7, Cat# 561479, BD biosciences). All samples were analyzed on a Becton-Dickinson LSR-II/Fortessa flow cytometer (BD biosciences, San Diego, CA) and analyzed by using Flowjo software (Tree Star Inc.).

### Lung viral titers

To determine lung viral loads, 10-fold serial dilutions (200 μl) of the lung homogenates were injected in the 10-day-old embryonated chicken eggs and incubated for 2 days. The allantoic fluids harvested after 2 days incubation were incubated with chicken red blood cells in 96-well V-bottom plates to determine hemagglutination activity as an indicator for virus replication. Lung viral titers were calculated by the Reed-Muench method [23].

### Statistics

Statistical significance was determined by using an unpaired two-tailed Student’s *t* test or one-way ANOVA. Results represent the means ± SEM (standard error of mean). Statistical analyses were performed using Prism software (* *P* <0.05, ** *P* <0.01, *** *P* <0.001).

## Results

### M2e5x VLP intranasal vaccination induces cross-reactive M2e- antibodies

Mucosal delivery of M2e5x VLP vaccine is expected to be effective in inducing protection against influenza virus respiratory pathogens. BALB/c mice (n = 10) were intranasally immunized with M2e5x VLP (15 μg) in a prime-boost strategy with a 4-week interval. Levels of antibodies specific for M2 peptides were low after prime, but boost immunization significantly increased M2 specific antibodies in sera (Fig 1B). M2e5x VLP vaccination resulted in similar amounts of IgG2a and IgG1 antibodies (Fig 1C), suggesting Th2 and Th1 immune responses. M2e5x VLP immune sera displayed low levels of cross reactivity to different heterosubtypic viruses A/California/2009 (A/Cal H1N1), A/Philippines/2/82 (A/Phil H3N2), and reassortant A/Vietnam/1203/2004 (A/Viet rgH5N1) (data not shown).

### Intranasal delivery of M2e5x VLP confers effective cross protection

Next, we assessed cross protective efficacy of M2e5x VLP intranasal vaccination. M2e5x VLP vaccinated mice were challenged with A/Philippines/2/1982 (A/Phil) (H3N2) or A/Viet rgH5N1 virus at 6 weeks after boost (Fig 2A). After A/Phil (H3N2) virus challenge, naïve mice showed a significant loss (approximately 25%) in body weight and died at day 8 or 9 (Fig 2B and 2C). In contrast, mice vaccinated with M2e5x VLP displayed 10% weight loss during 5 to 6 days and rapidly recovered body weight to a normal level by day 9 (Fig 2B). Infection of naïve mice with rgH5N1 resulted in over 17% loss in body weight, whereas M2e5x VLP immunized mice showed significantly less weight loss (2–3%) and fully recovered by day 6 after challenge (Fig 2D).

M2e5x VLP-immunized mice revealed a 14-fold decrease in virus titers compared to naïve mice with infection at 3 days after challenge (Fig 2E). Day 6 post challenge, lung viral loads in M2e5x VLP immune mice displayed approximately 76-fold lower than those in naïve mice. We also determined whether M2e5x VLP immunization induced M2-specific mucosal antibodies. Higher levels of M2-specific IgG and IgA antibodies were observed in the BAL fluids of immune mice than those in naïve mice before or after rgH5N1 virus challenge (Fig 2F and 2G). These results suggest that intranasal vaccination with M2e5x VLP induces M2-specific antibodies in sera and mucosal tissues, conferring effective cross-protection against heterosubtypic influenza viruses.

### M2e5x VLP intranasal vaccination prevents lung inflammatory responses after challenge

**Fig 3:**
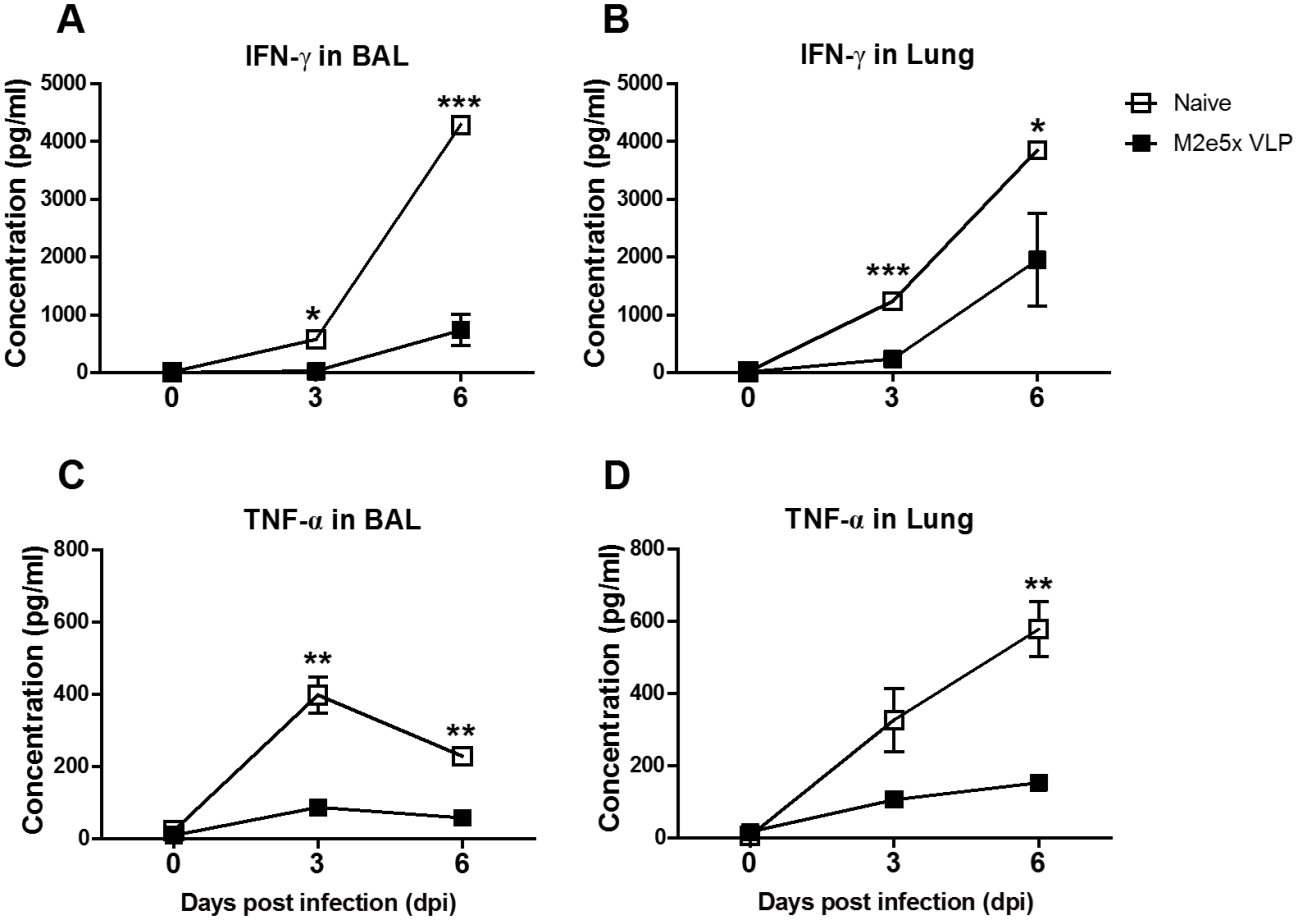
Vaccination with M2e5x VLP reduces inflammatory cytokines in the lungs after influenza virus challenge. Levels of inflammatory cytokines were determined day 3 and 6 post challenge with rgH5N1 (3×LD_50_) virus. Levels of IFN-γ **(A and B)** and TNF-α **(C and D)** were determined in the BALF and lung lysates by cytokine ELISA. Statistical significance was determined using an unpaired two-tailed Student’s *t* test. Error bars are means ± SEM of concentration or ratios from individual animals. *, *P*<0.05; **, *P*<0.01; ***, *P*<0.001.

Since over-production of cytokines causes severe pulmonary inflammatory responses during highly pathogenic influenza virus infection [24, 25], we determined the levels of IFN-γ and TNF-α. Naive mice were observed to secrete IFN-γ at day 3 and showed a dramatic increase in the levels of IFN-γ in the BAL and lungs day 6 post-infection (Fig 3A and 3B). In contrast, IFN-γ was not detected at day 3 and lower amounts of IFN-γ were observed in the BAL and lungs of M2e5x VLP immune mice day 6 post-infection (Fig 3A and 3B). In addition, the levels of TNF-α in the BAL and lungs from naïve mice were higher than those in immune mice day 3 and 6 post infection (Fig 3C and 3D). These data indicate that intranasal vaccination with M2e5x VLP effectively attenuates lung inflammatory cytokines after infection.

### M2e5x VLP vaccination reduces innate immune cell recruitment upon virus infection

**Fig 4:**
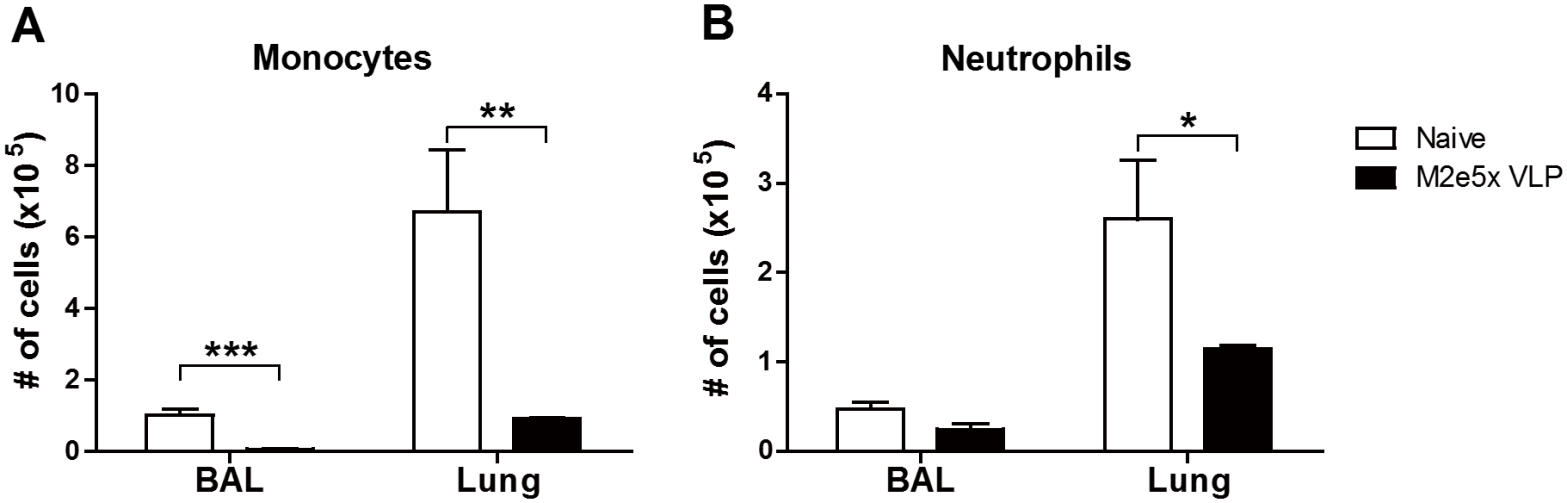
Mucosal vaccination with M2e5x VLP decreases innate immune cell recruitments after influenza virus infection. The cells were collected from BALF and lungs at 6 days after infection with rgH5N1 (3×LD_50_) virus. The number of neutrophils and monocytes from naïve and M2e5x VLP immune mice as gated on flow cytometry plots using markers including CD11b, Ly6c, and F4/80. **(A)** Inflammatory monocytes (CD11b^+^CD11c^-^Ly6c^hi^F4/80^+^) in the BAL and lungs. **(B)** Neutrophils (CD11b^+^CD11c^-^Ly6c^+^F4/80^-^) in the BAL and lungs. Statistical significance was determined using an unpaired two-tailed Student’s *t* test. Error bars indicate means ± SEM of concentration or ratios from individual animals. *, *P*<0.05; **, *P*<0.01; ***, *P*<0.001.

To better understand inflammatory cellular infiltration, we determined monocytes (CD11b^+^Ly6c^hi^F4/80^+^) and neutrophils (CD11b^+^Ly6c^+^F4/80^-^) in the lungs upon influenza virus infection. At 6 days after infection, significantly higher numbers of inflammatory monocytes were detected in the BAL and lungs from naive mice than those from M2e5x VLP immunized mice. (Fig 4A). In addition, a large number of infiltrating neutrophils from unimmune mice were observed in the lungs but not in the BAL compared to those in immune mice (Fig 4B). Overall, M2e5x VLP vaccination attenuates inflammatory innate cell infiltration into the lungs upon virus infection at 6 days after infection.

### M2e5x VLP vaccination enhances germinal center (GC) B cells and antibody-secreting cell responses

**Fig 5:**
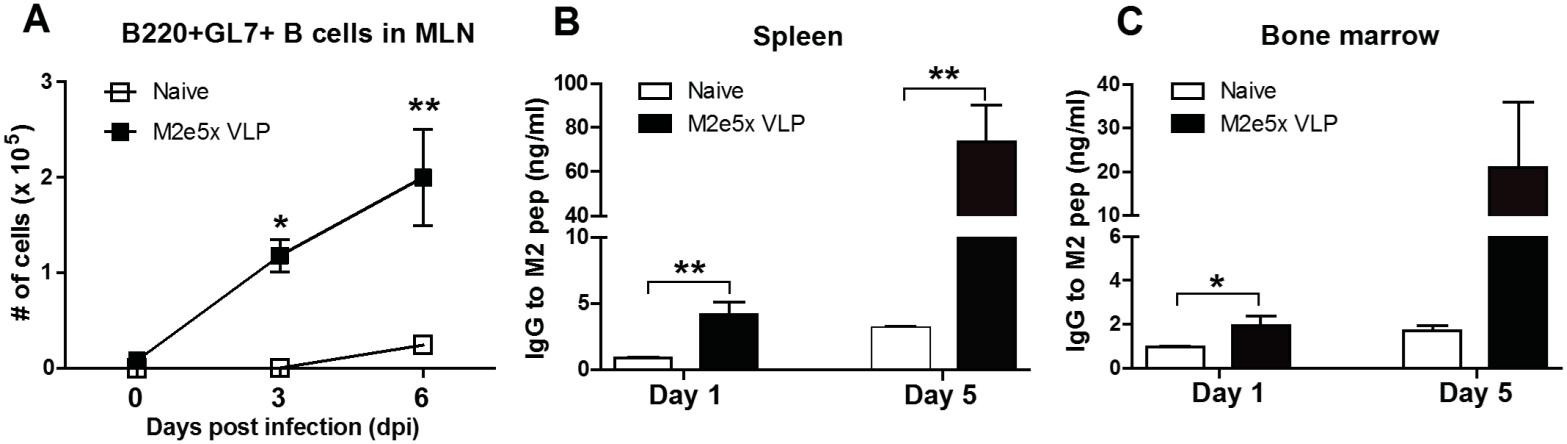
M2e5x VLP immunization enhances B cell activation and antibody secreting cell responses. The cells were harvested from the MLN **(A)**, spleen **(B)** and bone marrow **(C)** after challenge with rgH5N1 (3×LD_50_) influenza virus. **(A)** The numbers of B220^+^GL7^+^ germinal center B cells were analyzed by flow cytometry in the MLN of M2e5x VLP immune mice 3 and 6 days after challenge with rgH5N1 (3×LD_50_) influenza virus. The cells from the spleens **(B)** and bone marrows **(C)** were collected 6 days after challenge with rgH5N1 (3×LD_50_) influenza virus and cultured for 1 day or 5 days to detect IgG specific to M2e peptide. Statistical significance was determined using an unpaired two-tailed Student’s *t* test. Error bars are means ± SEM of concentration or ratios from individual animals. *, *P*<0.05; **, *P*<0.01.

We determined whether M2e5x VLP intranasal immunization would enhance B cell activation and M2e-specific antibody producing cell responses. GL7^+^ GC B cells (CD19^+^B220^+^) [26] were analyzed in the mediastinal lymph nodes (MLN) after challenge with rgH5N1. High numbers of GL7^+^ GC phenotypic B cells were observed in the MLN of immune mice compared to those in naïve mice day 3 and 6 post-infection (Fig 5A). To determine M2e-specific antibody secreting cell responses, the cells from the spleens and bone marrow were collected and cultured in the presence of M2e peptides. Significantly higher amounts of M2e specific antibodies were secreted from the splenocytes and bone marrow cells of M2e5x VLP immune mice compared to those in naïve mice during 1 to 5 days of cultures containing M2e peptides (Fig 5B and 5C). These data on GL7^+^ B cells and significant levels of *in vitro* antibody secreting cells suggest that M2e5x VLP intranasal vaccination is effective in inducing GC B cells and plasma cells.

### M2e5x VLP vaccination induces protective T cell responses

**Fig 6:**
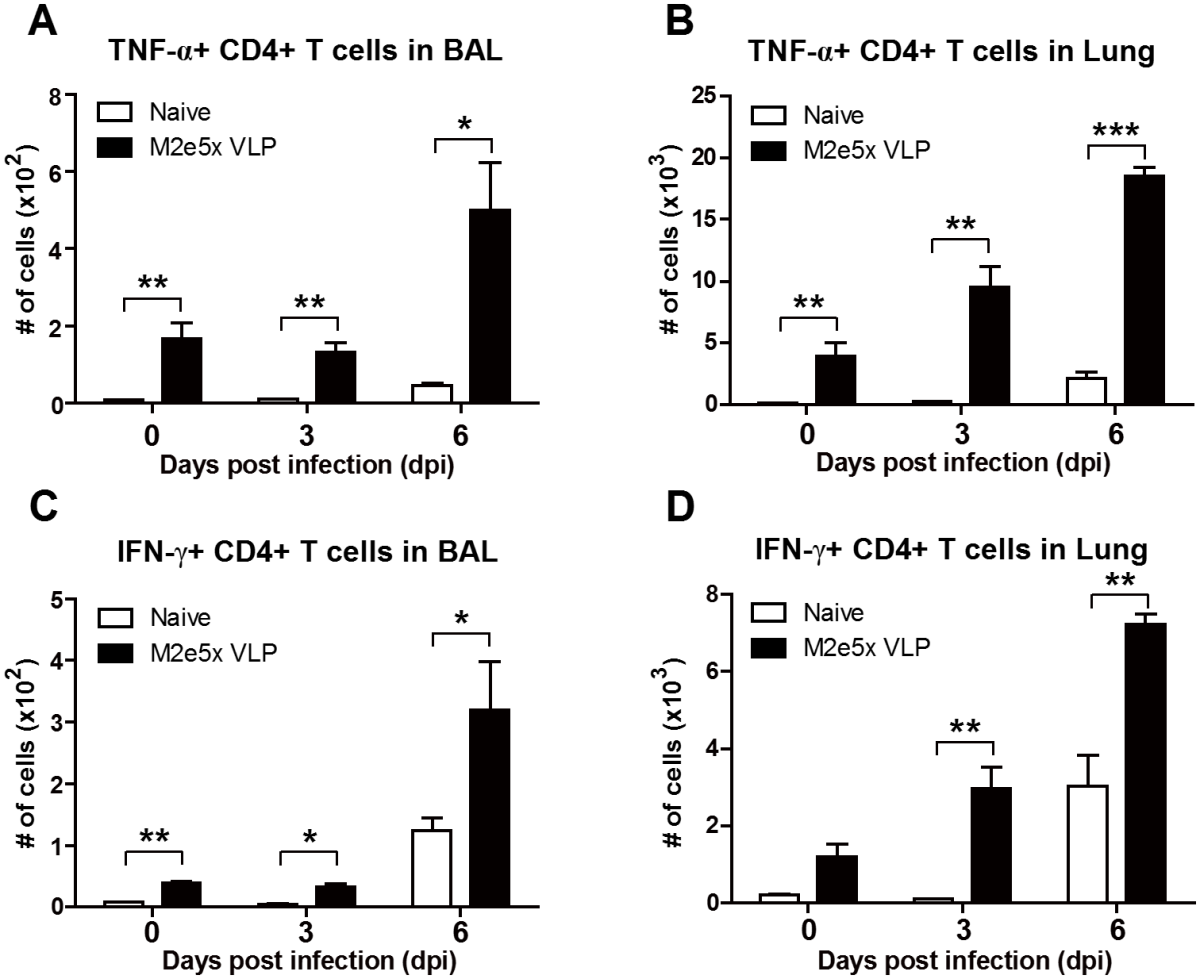
Mucosal immunization with M2e5x VLP enhances antigen-specific T cell responses after influenza virus infection. Immune cells were collected from the BAL and lungs of mice 3 and 6 days post infection with rgH5N1 (3×LD_50_) and cultured in the presence of M2 peptide as a stimulator. Antigen-specific T cells producing TNF-α **(A and B)** or IFN-γ **(C and D)** were analyzed by intracellular cytokine staining in the airways and lungs. Statistical significance was determined using an unpaired two-tailed Student’s *t* test. Error bars are means ± SEM of concentration or ratios from individual animals. *, *P*<0.05; **, *P*<0.01; ***, *P*<0.001.

To determine M2e-specific T cell responses, the cells harvested from the BAL fluids and lungs before and after influenza virus infection were stimulated with M2e peptides. Cytokine producing cellular responses were analyzed by an intracellular cytokine staining assay. TNF-α or IFN-γ producing CD4^+^ T cells were detected at higher levels in the BAL and lungs from the M2e5x VLP immune mice than those in naïve mice before challenge (day 0) and day 3 post-challenge (Fig 6). TNF-α or IFN-γ producing CD4^+^ T cells in the lungs of immune mice were increased in numbers (TNF-α: 2.4-fold; IFN-γ: 2.4-fold) day 6 post-challenge (Fig 6B and 6D). These results suggest that a low level of effector M2-specific T cells appears at the lung parenchymal tissues, which may be rapidly expanding upon virus infection.

**Fig 7:**
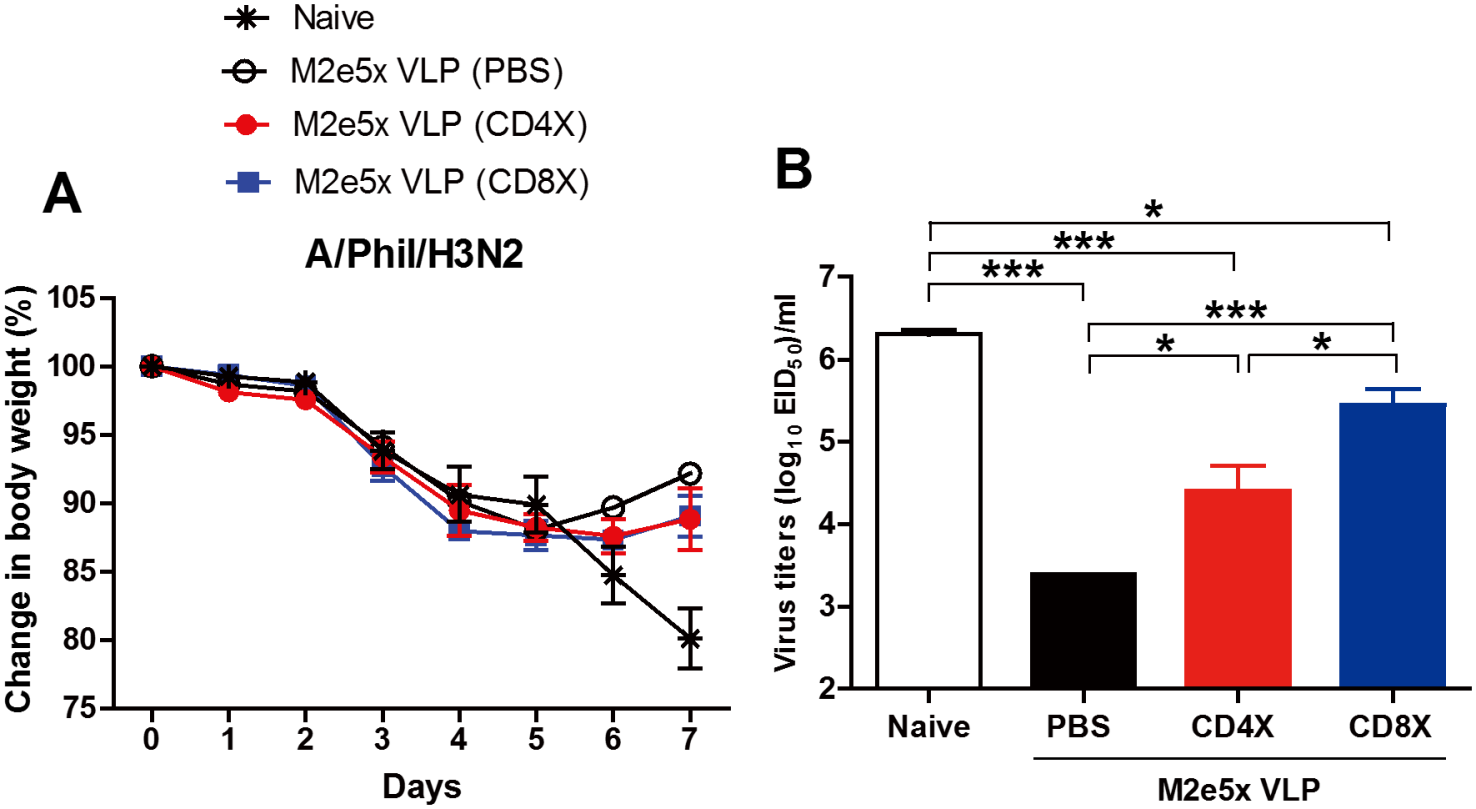
Roles of T cells in conferring protection after M2e5x VLP mucosal immunization. **(A)** Body weight changes. **(B)** Lung viral titers at day 6 post infection are presented as egg-infectious titers. At six weeks after boost immunization with M2e5x VLP, immune mice (n = 4) were intravenously received with anti-CD4 (CD4X) or anti-CD8 (CD8X) depletion antibodies at– 2 days and +1 day of challenge. PBS was used as a control. All mice were challenged with a lethal dose of A/Phil (H3N2) (5×LD_50_). Statistical significance was determined using one-way ANOVA or an unpaired two-tailed Student’s *t* test. Error bars are means ± SEM of concentration or ratios from individual animals. *, *P*<0.05; ***, *P*<0.001.

We further investigated the roles of CD4^+^ and CD8^+^ T cells in conferring protection. M2e5x VLP-vaccinated mice were administered PBS, CD4, or CD8 depletion antibodies before and during challenge with A/Phil (H3N2) virus (Fig 7). PBS-treated M2e5x VLP immune mice showed a better recovery in body weight than CD4- or CD8-depleted immune mice at 6 and 7 days after challenge (Fig 7A). Meanwhile, a substantial weight loss was observed in naïve mice day 6 and 7 post infection (approximately 15 and 20%, respectively). In line with body weight loss, highest levels of lung virus titers were detected in naive mice but M2e5x VLP immune mice that received with PBS showed lowest lung viral loads 7 days after infection (Fig 7B). As a result of CD4 depletion antibody treatment, lung viral loads significantly increased in immune mice compared to PBS-treated immune mice. Interestingly, CD8-depleted immune mice showed higher lung virus titers than CD4-depleted immune mice but lower than naïve mice, suggesting a role of CD8 T cells in protection by clearing lung viral loads in M2e5x VLP vaccinated mice (Fig 7B). These data suggest that intranasal vaccination with M2e5x VLPs induces cross protective T cell responses.

## Discussion

Cross protective cellular immune responses remain not well understood yet after mucosal vaccination. Here, we investigated the protective and immunological effects of intranasal vaccination with M2e5x VLPs. This study shows that intranasal vaccination with M2e5x VLP induces M2e-specific humoral and cellular immune responses including GC B cells, and plasma cells secreting M2e-specific antibodies as well as local CD4 and CD8 T cells likely contributing to cross protection. Therefore, intranasal vaccination with M2e5x VLP has the capacity to induce humoral and cellular immunity locally and systemically, which effectively confer cross protection against different serotypes of influenza viruses.

Many M2e-based vaccine studies were reported and evaluated in mice after systemic vaccination. Intranasal immunization was shown to be effective in inducing cross protection in mice [27, 28]. M2e protein (M2e5x) vaccines are a poor immunogen in the absence of adjuvants (data not shown). Various amounts of M2e vaccine doses (10 μg– 60 μg) in combination with different conjugate carriers and potent adjuvants were used to vaccinate mice [18, 29–33]. Nonetheless, M2e vaccines were not highly effective and M2e-vaccinated mice showed significant weight loss after challenge [7–14, 18, 29–33]. There are few studies on intranasal immunization with M2e vaccines (20–100 μg) in the presence of adjuvants [17, 18]. Protective efficacy of M2e vaccines was low and detailed cellular immune responses remain unknown.

Our previous study reported that intramuscular immunization with M2e5x VLPs in the absence of adjuvants induced M2 specific antibodies capable of conferring cross-protection [15]. Intramuscular prime immunization (10 μg of M2e5x VLP) induced significant levels of M2e antibodies as reported in our previous study [15]. In the current study, low levels of M2e antibodies were induced after intranasal prime immunization, which were significantly increased by boost vaccination (Fig 1B). It is also unclear how M2e5x VLP IM immune sera showed substantial levels of cross reactive antibodies to different viruses [15] whereas the intranasal immune sera displayed low levels of cross reactive antibodies (data not shown). Nonetheless, the levels of M2e specific antibodies after intranasal prime-boost vaccination (15 μg of M2e5x VLP) were found to be similar to those after IM prime-boost immunization (data not shown). It is expected that intranasal immune sera would exhibit *in vivo* protective effects since Intramuscular prime boost sera were shown to induce *in vivo* protection [15]. This study has focused on immune responses of B and T cells as well as on preventing inflammatory disease after intranasal immunization with M2e5x VLP and heterosubtypic influenza virus challenges. We found that intranasal immunization with M2e5x VLP was effective in inducing M2e specific IgG antibodies in sera systemically as well as IgG and IgA in BALF locally. Also, intranasal immunization with M2e5x VLP resulted in rapid expansion of GC GL7^+^ B cells in the draining MLN after challenge (day 0 to 6, Fig 5A), major sites of antigen drainage from the respiratory tracts [34]. In line with B cell expansion, an approximately 10-fold increase in M2e-specific IgG antibody levels during the 5 days of *in vitro* cultures suggest the induction of memory B cells that are rapidly differentiating into plasma cells secreting antibodies.

There seems to be a correlation between systemic disease of weight loss and levels of inflammatory cytokines (TNF-α, INF-γ) and cellular infiltrates (monocytes, neutrophils). This study suggests that intranasal immunization with M2e5x VLP induces effective cross protection. When C57BL/6 mice were intramuscularly vaccinated with M2e5x VLP in the presence of adjuvant (Alum+monophosphoryl lipid A [MPL]) and challenged, lower levels of interleukin (IL)-6 were detected in the vaccinated group than those in unvaccinated mice [35]. In the current study, BALB/c mice were intranasally vaccinated with M2e5x VLP without using any adjuvant. Pathogenic influenza virus infection causes severe lung inflammation characterized as proinflammatory cytokine storm and cellular infiltrates, which might lead to systemic disease of multi-organ failure [36, 37]. In response to inflammatory cytokines and chemokines, innate inflammatory cells such as neutrophils and monocytes as well as dendritic cells are recruited to the sites of viral infection, further contributing to the production of inflammatory cytokines. The naïve group showed high levels of lung viral titers at both 3 and 6 days after infection (Fig 2D), resulting in the production of high levels of inflammatory cytokines (Fig 3) probably due to infiltrating innate and adaptive immune cells in the lungs. The M2e5x VLP vaccine group showed better control of lung viral titers by approximately 100 folds, which is resulting in lessening the innate immune cellular infiltrates and reduction in the levels of inflammatory cytokines (IFN-γ, TNF-α). Therefore, better control of viral replication in the lungs by effective vaccination might have contributed to reducing the levels of inflammatory cytokines such as IFN-γ and TNF-α.

It may take time to induce antigen specific effector T cells (TNF-α^+^ and IFN-γ^+^ CD4 T cells) during viral infection of naïve mice even with high viral loads (Fig 6). The M2e5x VLP group showed a low level of vaccine-induced pre-existing antigen-specific TNF-α^+^ and IFN-γ^+^ CD4 T cells resident in the lung tissues and BAL before infection, which can secrete cytokines upon stimulation and rapidly expand during challenge infection (Fig 6). The presence of resident memory T cells in immune mice appears to attenuate inflammatory responses during influenza virus infection. Newly activated T cells are known to traffic to the lung parenchymal tissues from the draining lymph nodes [38]. IFN-γ or perforin expressing CD4 T cells were shown to enhance lung viral clearance and recovery [39, 40]. Expanded T cells in the M2e5x VLP group could have contributed to a better control of lung viral loads and a faster recovery from weight loss during a later stage of infection in addition to M2e antibodies, compared to the naïve mouse infection group (Fig 7). Better control of influenza virus loads will result in lowering the viral antigens available for activating antigen-specific T cells, which may also contribute to lowering the levels of inflammatory cytokines. The roles of CD4 and CD8 T cells were found to have similar effects on attenuating weight loss as evidenced by T cell depletion experiments. In contrast to the induction of CD4^+^ T cells producing cytokines upon stimulation with M2e peptides, mucosal vaccination with M2e5x VLPs induced low levels of M2e-specific cytokine producing CD8^+^ T cells, similar to those as detected in naïve mice after infection (data not shown). High viral loads in naïve mice led to the induction of CD8 T cells by day 7 after infection. Nonetheless, T cell immunity alone which was induced duringviral replication in an unimmunized condition was not sufficient to control viral loads and unable to prevent severe weight loss in the absence of M2e antibodies as observed in naïve mice after infection. Depletion of CD8 T cells has higher impact of increasing lung viral loads than CD4 T cell depletion in the M2e5x VLP vaccinated group, suggesting the important role of CD8 T cell immunity. It is possible that cytotoxic CD8 T cells secreting granzyme B are likely contributing to further reducing lung viral loads. These results provide evidence that both M2e humoral and cellular immunity play a critical role in conferring protection after intranasal vaccination of mice with M2e5x VLP.

## Supporting information

S1 Fig Efficacy of T cell depletion. Groups of mice (n = 3, BALB/c mice) were treated with CD4 T cell depleting antibody (200 μg in 200 μl, Clone GK1.5), IgG isotype control (Clone LTF-2, rat IgG2b), or buffer (PBS) two times (days 0 and 2). At day 7, the T cell levels were determined in bloods by flow cytometry.
(PDF)
