# Supplementary material for: Intranasal vaccination with M2e5x virus-like particles induces humoral and cellular immune responses conferring cross-protection against heterosubtypic influenza viruses
Source: PLoS One. 2018 Jan 11;13(1):e0190868. doi: 10.1371/journal.pone.0190868 (PMC5764335; doi:10.1371/journal.pone.0190868)
Supplement: S1 Fig — Groups of mice (n = 3, BALB/c mice) were treated with CD4 T cell depleting antibody (200 μg in 200 μl, Clone GK1.5), IgG isotype control (Clone LTF-2, rat IgG2b), or buffer (PBS) two times (days 0 and 2). At day 7, the T cell levels were determined in bloods by flow cytometry. (PDF) [file pone.0190868.s001.pdf]

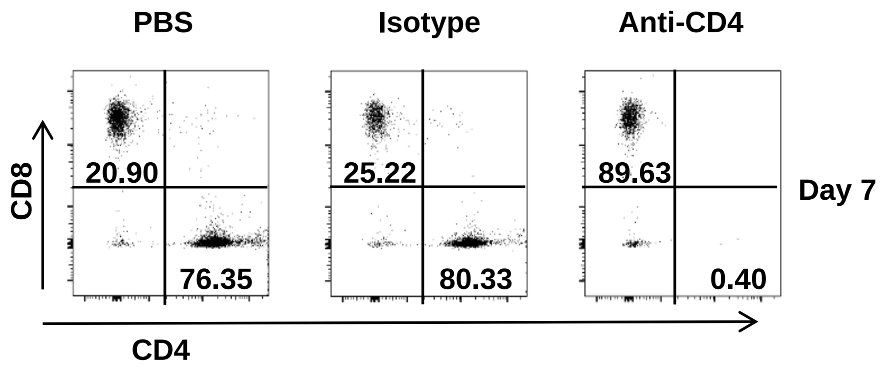

Supplementary Figure 1. Efficacy of T cell depletion. Groups of mice (n=3, BALB/c mice) were treated with CD4 T cell depleting antibody (200 µg in 200 µl, Clone GK1.5), IgG isotype control (Clone LTF-2, rat IgG2b), or buffer (PBS) two times (days 0 and 2). At day 7, the T cell levels were determined in bloods by flow cytometry.
